# Effort-Reward Imbalance at Work and Overcommitment in Patients with Acute Myocardial Infarction (AMI): Associations with Return to Work 6 Months After AMI

**DOI:** 10.1007/s10926-020-09942-7

**Published:** 2020-11-16

**Authors:** Sarah Ruile, Christine Meisinger, Katrin Burkhardt, Margit Heier, Christian Thilo, Inge Kirchberger

**Affiliations:** 1 Chair of Epidemiology, UNIKA-T Augsburg, Ludwig-Maximilians-Universität München, Neusässer Str. 47, 86156 Augsburg, Germany; 2 Institute for Medical Information Processing, Biometry and Epidemiology-IBE, Ludwig-Maximilians-Universität München, Munich, Germany; 3 Pettenkofer School of Public Health, Munich, Germany; 4 MONICA/KORA Myocardial Infarction Registry, University Hospital of Augsburg, Augsburg, Germany; 5 Independent Research Group Clinical Epidemiology, Helmholtz Zentrum München, German Research Center for Environmental Health, Neuherberg, Germany; 6 Department of Laboratory Medicine and Microbiology, University Hospital of Augsburg, Augsburg, Germany; 7 Institute of Epidemiology, Helmholtz Zentrum München, German Research Center for Environmental Health, Neuherberg, Germany; 8 KORA Study Centre, University Hospital of Augsburg, Augsburg, Germany; 9 Department of Internal Medicine I – Cardiology, University Hospital of Augsburg, Augsburg, Germany; 10 Centro de Investigación Biomédica en Red, Enfermedades Cardiovasculares (CIBERcv), Madrid, Spain

**Keywords:** Myocardial infarction, Occupational stress, Return to work

## Abstract

*Purpose* Stress-related factors influence the adaptation to life after acute myocardial infarction (AMI), including return to work. The goal of this study was to investigate the effect of work-related stress, (expressed by the effort-reward imbalance (ERI) model) on return to work after AMI. *Methods* A longitudinal study with AMI patients was conducted in order to assess associations between the independent variables effort, reward, ERI and overcommitment and the outcome return to work after AMI. Return to work was inquired at 6 months follow-up. Logistic regression models were applied in the analysis. The fully-adjusted model included demographic, clinical, social, stress-related and health-related quality of life (HRQOL) covariables. *Results* Of the 346 enrolled patients aged 31 to 82 years, 239 (69.1%) were included in the regression analysis. In the unadjusted model ERI presented an odds ratio (OR) of 1.72 (95% confidence interval (CI) 0.86–3.42). Associations for effort and overcommitment were 0.98 (95% CI 0.83–1.15) and 1.09 (95% CI 0.99–1.18). However, reward showed a significantly inverse association with return to work with an OR of 0.90 (95% CI 0.83–0.99). In the fully adjusted model the OR of ERI decreased to 1.20 (95% CI 0.49–2.96). Effort, reward and overcommitment also showed attenuated ORs without significant results in all models. Diabetes mellitus, current smoking, low physical and low mental HRQOL presented significantly negative relations with return to work. *Conclusions* Work-related stress appears less important than HRQOL and resilience in terms of return to work after AMI.

## Introduction

Cardiovascular diseases (CVD) are one of the leading causes of death worldwide [1]. For the European population ischemic heart diseases represent one of the biggest subgroups of CVD and especially acute myocardial infarction (AMI) has a high mortality rate [2–4]. Besides, survivors of an AMI often have to cope with impaired physical and mental health-related quality of life (HRQOL) [5, 6].

A number of personal and environmental factors may contribute to the adaptation to life after AMI. For instance, stress was shown to have an adverse effect on the life post-AMI. It is associated with a significantly higher 2-year mortality, physical limitations and worse HRQOL [7, 8]. Particularly, stress at work was found to significantly increase the risk to develop cardiovascular diseases up to a hazard ratio (HR) of 3.13 [9]. A well-known model of stress at work is the effort-reward imbalance (ERI) model, introduced by Siegrist et al. in 1996 [10, 11]. It compares the efforts applied regarding work, like workload or numerous interruptions, with the received rewards [12]. High effort and low reward cause a negative disparity and negative work-related stress. Effort and reward are extrinsic factors. A separate component and intrinsic factor is overcommitment defined as the individual trait for high willingness regarding work-related effort [12, 13].

Studies have demonstrated that ERI at work is a relevant risk factor for the development of an AMI [9] and overcommitment is associated with cardiovascular diseases such as hypertension or coronary atherosclerosis [14, 15]. Although associations were found between high ERI and recurrent coronary heart disease events after AMI [16], little knowledge exists about the relation between pre-AMI ERI and overcommitment, and life after an AMI.

In particular, ERI at work and overcommitment may affect return to work which is an important indicator of disease recovery [17]. Failure to return to work has a wide range of negative consequences on the affected individuals including physical and psychological discomfort [18, 19], increased depression [20], impaired quality of life [21] and life satisfaction [22], and increased financial burden on patients and families [23]. Besides individual consequences, work disability also imposes considerable societal costs [24].

The objective of this study is to determine the association between pre-AMI ERI at work and return to work 6 months post AMI. Another goal is to explore the association between overcommitment and return to work.

## Methods

### Study Design

A longitudinal observational study was carried out. The study population consisted of patients with AMI admitted to a hospital in the study region of the MONICA/KORA (Monitoring Trends and Determinants in Cardiovascular Diseases/Cooperative Health Research in the Region of Augsburg) Myocardial Infarction Registry, Germany [25].

In March 2014, a pilot phase with ten patients was carried out to confirm the feasibility of the baseline questionnaires and to test the study processes. In the main study, patients with diagnosed AMI who were enrolled in study, filled in a questionnaire during their hospital stay. A postal survey was sent to all participants 6 and 12 months after hospital discharge. The study was carried out from April 2014 to June 2017. Written informed consent was provided by all participants. Ethical approval for the study was obtained from the ethics committee of the Bavarian Medical Association (No. 14007).

The present paper reports a secondary data analysis restricted to baseline and 6 months follow-up data.

### Study Population

Seven hospitals from the study region of Augsburg and two in the adjacent counties provided data on admitted patients. In order to identify suitable participants, medical records of hospitalized patients were screened. If patients met all inclusion criteria, namely confirmed AMI, a regular paid employment of at least 10 h per week, sufficient knowledge of the German language and informed written consent, they were enrolled in the study. Persons with lacking German language skills were excluded since a number of the used questionnaires were not available in all necessary languages.

### Survey Data

The baseline survey included the following questionnaires to collect information on the study outcome and possible confounders.

#### Effort-Reward-Imbalance-Scale (ERI)

The dependent variables of this study were Effort-Reward-Imbalance (ERI) and overcommitment, measured by the German version of the short-form Effort-Reward-Imbalance Questionnaire. The underlying theoretical model assumes that stress at work is made up by a ratio of the given obligations of somebody and the received rewards, i.e. salary or reduction of work load [26]. The two components are influenced by overcommitment, which is defined as a form of overestimation of one’s own capacity.

The questionnaire consists of 23 4-point Likert-scaled questions allocated to two subscales, effort (3 questions, range 3 to 12), reward (7 questions, range 7 to 28), and the overcommitment scale (6 questions, range 6 to 24). The effort and reward subscales result in the ERI-score with a possible range from 0.25 (low level of ERI) to 3.99 (high level of ERI) [26]. The ERI questionnaire was already used in a few studies with AMI patients and showed significant associations with the occurrence of stress-related diseases [9, 16]. Moreover, it was validated in Germany and demonstrated sufficient internal consistency [10].

#### Short-Form 36 Health Survey (SF-36)

The German version of the SF-36 was used to measure HRQOL in the study population [27]. It consists of 8 subscales that can be summarized in a physical and mental summary score, ranging from 0 to 100. This questionnaire has been tested in numerous studies including studies on patients with AMI [6].

#### Resilience Scale (RS-11)

The Resilience Scale (RS-11) gathers information about personal resilience, containing 11 items [28]. The total score ranges from 11 (low resilience) to 77 (high resilience). It has already been validated in Germany and applied in AMI patients [28].

#### Perceived Stress Scale (PSS4)

The PSS4 was used to measure overall perceived stress by applying four questions. The summary score ranges from 0 to 16 with higher scores indicating more perceived stress. It has already been used in studies with AMI patients [7] and a validated German version is available [29].

#### Questionnaire on Social Support (F-SozU)

The Questionnaire on Social Support was selected to gather information on received social support [30]. The 14 questions of the short form showed good psychometric item properties, as well as a very acceptable reliability [31]. F-SozU scores range from 0 (low social support) to 14 (high social support).

Furthermore, four self-developed questions addressing stress at work, stress at home, and financial burden with 3- to 5-point Likert-scales were applied. Finally, information on age, sex, marital status, type of employment and the amount of hours worked per week was collected.

To assess the study outcome, return to work after AMI, patients were asked in the 6 months follow-up whether they have returned to work and what were the reasons in case of no return to work.

### Clinical Data

Health characteristics such as body mass index (BMI), AMI risk factors, AMI type, co-morbidities and medical treatment (pre- and in-hospital) as well as invasive treatment were obtained by patient interview and extracted from medical records.

### Data Collection

For the data collection of the baseline survey, trained study nurses (registered nurses) got in touch with in-hospital patients at the wards and handed out information to the potential participants. After receiving the informed consent from the patients, they asked the participants to complete the questionnaires. The postal follow-up was sent to the participants 6 months after discharge from the hospital. If participants did not return the documents, they were reminded by telephone by the study nurse. In case no phone number was recorded, a postal reminder was sent.

### Statistical Analysis

G*Power 3.1 program was used to perform sample size estimation [32]. At a two-sided type 1 error level of 5% with an effect size of 0.3 and 80% power at least 240 patients are needed for a regression model including 10 covariables. Since former studies within the MONICA/KORA Myocardial Infarction Registry showed that about 30% of included patients might be lost to follow-up or die, 343 patients should be included in the present study.

Descriptive statistics were performed and in order to identify significant differences between the groups of return to work and no return to work either Chi squared or Fisher’s F-test were applied to categorical variables (sex, marital status, worker type, BMI, CVD risk factors, co-morbidites, AMI classification, cardiac arrest, stress at home, financial burden, working hours per week, stress at work) and either Student’s T-test or Wilcoxon rank sum test were applied to continuous variables (age, resilience, social support, perceived stress, HRQOL, ERI) [33]. Similar methods were used to compare the characteristics of people with missing responses to people without missing responses.

Logistic regression was performed to determine the association between ERI, effort, reward and overcommitment, and return to work. Return to work was the dependent variable in all models. Independent variables were ERI, effort, reward and overcommitment.

Possible covariables were selected by using directed acyclic graphs (DAG) [34]. A DAG displays assumptions about the relationship between variables based on available literature. It helps to describe causal pathways and to identify confounding, colliding and mediating variables. According to the DAG performed for the present study age, sex, diabetes, smoking, HRQOL, perceived stress, social support, and resilience were identified to be related both with the independent variables (ERI, effort, reward, overcommitment) and the outcome (return to work), such as sex and social support, or with the outcome in biasing or causal pathways.

For each of the independent variables (ERI, effort, reward, overcommitment) an unadjusted model and a model adjusted for sex and age was calculated. Moreover, a fully adjusted model for the independent variable ERI was calculated which included age, sex, diabetes, smoking, hours worked per week, physical and mental HRQOL, perceived stress, social support, resilience and overcommitment. The association between the independent variables effort, reward, overcommitment and the dependent variable return to work was also determined in a fully adjusted model considering the covariables mentioned above. Akaike’s Information Criterion (AIC) was used to select the best model [35]. The level of significance was set to p < 0.05 for all analyses. Variance inflation and the interaction effect of age and sex were tested. Finally, for each independent variable, the estimates derived from the unadjusted models, the model adjusted for age and sex and the fully adjusted model were compared.

As a sensitivity analysis, logistic regression analyses (unadjusted, adjusted for age and sex, fully adjusted) were performed for the subgroup of AMI patients, who did not return to work because of medical certified sickness.

Statistical analyses were conducted with SAS University Edition.

## Results

In the study period, 1735 persons with AMI were admitted to the recruiting hospitals and were contacted by a study nurse. 1230 were not eligible because they had no regular paid employment of at least 10 h per week before the AMI and 30 were lacking sufficient knowledge of the German language. From the 475 eligible patients, 127 (26.7%) refused participation. For the data analysis, two patients were excluded because the AMI diagnosis was withdrawn and three due to non-completion of the baseline ERI questionnaire, respectively. From 343 patients left, 286 (83.4%) reported whether they returned to work or not 6 months post AMI. Compared with participants with available information on return to work (n = 239), participants with missing information on return to work (n = 47) were significantly more likely to be female (29.8% vs. 13.8%) and to have a history of diabetes (29.8% vs. 15.2%). In addition, they were more likely to have permanent stress at work (44.4% vs. 26.4%) and to have severe financial burden (31.9% vs. 16.7%). HRQOL scores were significantly worse, whereas scores of overall perceived stress and overcommitment were higher.

### Sample Characteristics

Sample characteristics derived from the baseline survey are shown in Table 1, for the overall sample as well as stratified by return to work. The sample of 286 AMI patients was mostly male (83.6%) and married (76.1%) with a mean age of 54.1 years. Cardiac risk factors such as current smoking, hypertension or hyperlipidemia were present in about half of the sample, whereas obesity (BMI > 30 kg/m^2^), diabetes mellitus, previous myocardial infarction (MI) or stroke, coronary heart disease and angina pectoris were less prevalent. Almost half of the AMI patients were white-collar employees and reported moderate stress at work with a slightly increased level of ERI (1.23).

**Table 1:** Sample characteristics at baseline, overall and stratified by return to work

	Total sample	Return to work		p value
	N = 286^a^	Yes n = 236 (82.52)	No n = 50 (17.48)	
Sex				
Male	239 (83.57)	203 (86.02)	36 (72.00)	.0015^b^
Female	47 (16.43)	33 (13.98)	14 (28.00)	
Age, *mean (SD*^*c*^*)*	54.10 (7.58)	53.86 (7.34)	55.26 (8.65)	.2447^d^
Marital status				
Married	188 (76.11)	157 (76.96)	31 (72.09)	.4963^b^
Not married	59 (23.89)	47 (23.04)	12 (27.91)	
Worker type				
Blue collar worker	97 (34.04)	76 (32.34)	21 (42.00)	
White collar worker	136 (47.72)	113 (48.09)	23 (46.00)	.4495^e^
Self-employed	51 (17.89)	45 (19.15)	6 (12.00)	
Others	1 (0.35)	1 (0.43)	0 (0.00)	
BMI (kg/m^2^)				
BMI^f^ ≤25	60 (20.98)	53 (22.46)	7 (14.00)	.2817^b^
BMI > 25–≤ 30	149 (52.10)	123 (5212)	26 (52.00)	
BMI > 30	77 (26.92)	60 (25.42)	17 (34.00)	
CVD risk factors and co-morbidity				
Hypertonus, yes	163 (57.39)	134 (57.26)	29 (58.00)	.9240^b^
Hyperlipidemia, yes	138 (48.76)	110 (47.21)	28 (56.00)	.2592^b^
Ex-smoker	93 (32.98)	78 (33.62)	15 (5.32)	.4343^b^
Current smoker	136 (48.23)	108 (46.55)	28 (56.00)	
Diabetes mellitus, yes	50 (17.61)	35 (14.96)	15 (30.00)	.0112^b^
Coronary heart disease, yes	43 (15.14)	34 (14.53)	9 (18.00)	.5344^b^
Angina pectoris, yes	19 (6.74)	12 (5.17)	7 (14.00)	.0543^e^
Previous myocardial infarction, yes	35 (12.32)	28 (11.97)	7 (14.00)	.6912^b^
Previous apoplex, yes	7 (2.46)	7 (2.99)	0 (0.00)	.6106^e^
COPD^g^, yes	11 (3.89)	8 (3.43)	3 (6.00)	.4171^e^
Heart failure, yes	4 (1.41)	3 (1.28)	1 (2.00)	.5412^e^
Renal insufficiency, yes	7 (2.46)	7 (2.99)	0 (0.00)	.6106^e^
Classification of infarction				
STEMI^h^	127 (46.18)	105 (46.05)	22 (46.81)	.3461^e^
NSTEMI^i^	135 (49.09)	114 (50.00)	21 (44.68)	
Bundle branch block	13 (4.73)	9 (3.75)	4 (8.51)	
Clinical factors				
Cardiac arrest preclinical, yes	13 (5.35)	9 (4.48)	4 (9.52)	.2481^e^
Health-related quality of life, *mean (SD)*				
General health perception	61.17 (18.44)	62.41 (18.08)	55.34 (19.18))	.0166^d^
Mental health	68.32 (19.85)	70.96 (18.57)	58.98 (21.14)	< .0001^d^
Bodily pain	65.16 (33.25)	69.39 (31.79)	45.18 (33.00)	< .0001^d^
Physical functioning	62.21 (29.46)	65.87 (27.63)	45.00 (31.90)	< .0001^d^
Emotional role functioning	74.20 (39.54)	80.56 (34.91)	42.55 (45.95)	< .0001^d^
Physical role functioning	58.98 (42.92)	63.14 (41.41)	39.50 (44.92)	.0006^d^
Social role functioning	76.27 (25.41)	78.71 (23.28)	64.75 (31.51)	.0037^d^
Vitality	52.05 (22.91)	55.55 (20.78)	35.60 (25.37)	< .0001^d^
Physical sum score	41.91 (10.66)	43.10 (10.09)	36.12 (11.57)	.0002^d^
Mental sum score	49.05 (11.73)	50.62 (10.63)	41.38 (13.79)	< .0001^d^
Resilience, *mean (SD)*	61.72 (9.54)	61.88 (9.07)	60.96 (11.60)	.8447^d^
Social Support, *mean (SD)*	4.52 (0.84)	4.56 (0.83)	4.34 (0.856)	.5586^d^
Perceived stress, *mean (SD)*	6.01 (2.96)	5.70 (2.80)	7.46 (3.32)	.0006^d^
Stress at home				
Never/rarely	162 (56.64)	134 (56.78)	28 (56.00)	.1146^b^
Sometimes	111 (38.81)	94 (39.83)	17 (34.00)	
Always	13 (4.55)	8 (3.39)	5 (10.00)	
Financial burden				
Never/rarely	133 (46.50)	115 (48.73)	18 (36.00)	.0784^b^
Moderate	98 (34.27)	81 (34.32)	17 (34.00)	
Severe	55 (19.23)	40 (16.95)	15 (30.00)	
Work factors				
Hours worked per week ≥35	240 (84.21)	204 (86.44)	36 (73.47)	.0235^b^
Hours worked per week ≤34 h	45 (15.79)	32 (13.56)	13 (26.53)	
Stress at work				
Never/rarely	54 (19.01)	47 (19.92)	7 (14.58)	.1124^b^
Sometimes	147 (51.76)	126 (53.39)	21 (43.75)	
Always	83 (29.23)	63 (26.69)	20 (41.67)	
Effort-reward imbalance, *mean (SD)*				
Effort	8.74 (2.18)	8.74 (2.19)	8.74 (2.15)	.9884^d^
Reward	17.94 (3.66)	18.18 (3.53)	16.87 (4.10)	.0572^d^
Overcommitment	15.35 (3.98)	15.10 (3.76)	16.52 (4.73)	.0168^d^
ERI	1.23 (0.46)	1.20 (0.44)	1.34 (0.54)	.0744^d^

^a^Values are expressed as numbers (percentage) unless otherwise indicated. Denominator may vary because of missing information

^b^Chi squared test

^c^Standard deviation

^d^Wilcoxon–Mann–Whitney-test

^e^Fisher’s exact test

^f^Body mass index

^g^Chronic obstructive pulmonary disease

^h^ST elevation myocardial infarction

^i^Non-ST elevation myocardial infarction

From the 286 AMI patients, 236 (82.5%) returned to work at follow-up, whereas 50 (17.5%) didn’t. Most of those not returning to work were certified sick (n = 32, 71.11%). Other reasons were unemployment (n = 6, 13.33%), partial pension (n = 5, 8.89%), occupational retraining (n = 1, 2.22%), housekeeping (n = 1, 2.22%), early retirement (n = 1, 2.22%) and pension (disability, age) (n = 1, 2.22%). People who returned to work were about 1.5 years younger than participants, who didn’t return to work. A significant difference was found in the sex variable, where 36 (15%) of the male people and 14 (30%) of the female people didn’t return to work. Regarding cardiac risk factors and clinical determinants only diabetes showed a significant difference between the two groups, with higher prevalence in patients who didn’t return to work. Furthermore, all mean scores of the SF-36 subscales were significantly lower in the group with no return to work after AMI, including the physical and mental summary score. The group with no return was significantly more likely to work 34 h per week or less before the AMI compared with those who returned to work. AMI patients with no return to work had higher scores of overcommitment than patients with return to work. Also, the group with no return to work reported significantly more overall stress compared with the other group.

### Association Between ERI and Overcommitment, and Return to Work

Logistic regression analysis was conducted on 239 (69.7%) patients with complete information on all covariables at 6 months follow-up.

In the unadjusted logistic regression model (Table 2) an odds ratio (OR) of 1.72 (95% confidence interval (CI) 0.86–3.42) for ERI was observed, implying a greater chance of no return to work in patients with high ERI scores. Statistical significance was not accomplished, but the reward subscale and overcommitment showed significant results with a greater chance of high reward scores in AMI patients, who returned to work and a greater chance of high overcommitment scores in patients, who did not return to work.

**Table 2:** Chance of no return to work 6 months post myocardial infarction

Independent variable	Unadjusted			Adjusted for sex and age		
	OR^a^	95% CI^b^	p value	OR	95% CI	p value
ERI^c^	1.72	0.86–3.42	.1253	1.65	0.82–3.31	.1571
Effort	0.98	0.83–1.15	.7826	0.98	0.83–1.15	.8149
Reward	**0.90**	**0.83–0.99**	**.0339**	**0.91**	**0.83–0.99**	**.0448**
Overcommitment	**1.09**	**0.99–1.18**	**.0060**	1.08	0.99–1.18	.0699

Significant results (p < 0.05) are highlighted in bold

Logistic regression models (n = 239)

^a^Odds ratio

^b^Confidence interval

^c^Effort-reward Imbalance

After adjustment for age and sex the OR of ERI changed to 1.65 (95% CI 0.82–3.31), slightly smaller than the OR in the unadjusted model and still not significant. The OR of reward and overcommitment hardly changed, but only reward showed statistical significance.

The fully adjusted model for ERI, including all relevant covariates selected by AIC, is shown in Table 3. The association of ERI with no return to work decreased to an OR of 1.20 (95% CI 0.49–2.96) in comparison to the unadjusted and sex- and age adjusted models, but still showed a small positive association without statistical significance. The covariables DM and current smoking showed a relatively high OR (> 2.5) for no return to work without reaching statistical significance. In contrast, low physical and mental HRQOL were significantly associated with no return to work in AMI patients. Overcommitment showed no significant relation with return to work in the fully adjusted model.

**Table 3:** Association between effort-reward-imbalance (ERI) and no return to work 6 months post myocardial infarction

	OR^a^	95% CI^b^	p value
ERI	1.20	0.49–2.96	.6917
Age	1.03	0.97–1.10	.2969
Sex^c^	1.05	0.33–3.38	.9352
Diabetes mellitus	2.59	0.96–6.99	.0601
Smoking (current)^d^	3.46	0.99–12.05	.0516
Smoking (former)^d^	1.25	0.32–4.97	.7508
Hours worked per week (full time)^e^	0.51	0.15–1.74	.2801
Physical HRQOL^f^	**0.96**	**0.92–0.99**	**.0252**
Mental HRQOL^f^	**0.94**	**0.90–0.98**	**.0022**
Social support	1.25	0.74–2.12	.3968
Perceived stress	1.14	0.96–1.34	.1271
Resilience	1.04	0.99–1.10	.0819
Overcommitment	0.99	0.87–1.11	.8217

Significant results (p < 0.05) are highlighted in bold

Multivariable logistic regression model (n = 239)

^a^Odds ratio

^b^Confidence interval

^c^Reference: female

^d^Reference: never smoker

^e^Reference: part-time

^f^Health-related quality of life

Table 4 illustrates the results of the fully-adjusted model with the ERI subscales effort and reward and overcommitment. Lower amounts of effort and reward had a slightly higher but not significant chance of no return to work in AMI patients. In common with the previous fully-adjusted model, DM and smoking also had high but not significant OR’s of no return to work. In addition, the AMI patients showed significantly lower chances of no return to work in case of lower physical and mental HRQOL scores. A high level of resilience was associated with a significantly higher odds of no return to work.

**Table 4:** Association between effort, reward and overcommitment and no return to work 6 months post myocardial infarction

	OR^a^	95% CI^b^	p value
Effort	0.87	0.69–1.09	.2213
Reward	0.92	0.83–1.03	.1555
Overcommitment	1.02	0.90–1.16	.7693
Age	1.03	0.97–1.09	.3924
Sex^c^	0.93	0.28–3.14	.9063
Diabetes mellitus	2.48	0.90–6.81	.0777
Smoking (current)^d^	3.25	0.94–11.21	.0626
Smoking (former)^d^	1.15	0.29–4.58	.8451
Hours worked per week (full time)^e^	0.69	0.19–2.53	.5726
Physical HRQOL^f^	**0.95**	**0.91–0.99**	**.0207**
Mental HRQOL^f^	**0.94**	**0.90–0.98**	**.0021**
Social support	1.30	0.78–2.18	.3169
Perceived stress	1.13	0.96–1.34	.1428
Resilience	**1.05**	**1.01–1.10**	**.0466**

Significant results (p < 0.05) are highlighted in bold

Multivariable logistic regression model (n = 239)

^a^Odds ratio

^b^Confidence interval

^c^Reference: female

^d^Reference: never smoker

^e^Reference: part-time

^f^Health-related quality of life

Variance inflation factor scores were below 1.85 showing no multicollinearity among the covariables.

### Sensitivity Analysis

In order to confirm the stability of the regression models, the models were recalculated for persons with medical certified sickness as the reason for no return. The sample size was 226, with 29 (12.8%) AMI patients not returning to work. Compared to the models with full sample size for return to work, a slightly higher OR of ERI was found in the unadjusted model (OR = 1.87 (95% CI 0.84–4.17) vs. 1.72 (0.86–3.42)). The same effect was seen in the model adjusted for sex and age. Effect sizes of effort, reward and overcommitment did not considerably differ in these two models, compared to the models with full sample size. In the fully adjusted model, the effect sizes of ERI and the variable on hours worked per week, were smaller with an OR of 1.00 (95% CI 0.35–2.86) and 0.85 (95% CI 0.19–3.80), respectively. An opposite effect was found regarding current smoking, which showed an increased OR of 5.6 (95% CI 0.99–31.89).

## Discussion

Our study results indicated associations between ERI, effort, reward and overcommitment and return to work after AMI, which achieved statistical significance for reward and overcommitment in the unadjusted regression models. After adjustment for possible covariates, associations of high ERI, high effort, low reward and high overcommitment with no return to work remained but were not significant anymore.

In the present study, rates of return to work 6 months after AMI (82.5%) were consistent with the results from Smedegaard et al. [36], who reported that about 80% of people with AMI returned after 6 months. In other studies with AMI patients, rates of return to work ranged between 55.9% and 91.1% 1 year after AMI [37, 38]. Thus, comparability of rates of return to work was restricted due to the differing follow-up points of time.

Studies, which investigated work-stress in patients with CVD had considerable variations in terms of patient population, study design, assessment of work-stress and outcomes. A previous study found associations of high ERI and incidence of coronary heart disease events, as well as associations between high ERI and recurrent heart disease events [16]. Also, associations of low reward regarding no return to work were seen in this study.

Stress at work, ascertained with the Copenhagen Psychosocial Questionnaire (COPSOQ), was associated with sickness absence 3 months after interventional treatment of patients with coronary heart disease [39]. However, these effects were found in a cohort of patients with a high risk for AMI, but without current AMI.

Associations of ERI with expected time for return to work were investigated in a cross-sectional study by Soederberg et al. in 509 persons with acute coronary syndrome [40]. They conducted linear regression models with job strain and ERI as independent variables. In the overall sample a higher chance of delayed return to work was found in participants with high ERI, with an OR of 3.00 (β = 1.1) in the unadjusted model. After adjustment for occupational status, self-efficacy and general mental health, the effect estimate decreased but remained significant. The average ERI score was 0.6, being considerably lower than the average ERI score of the present study (1.34). Compared to the present study, a different definition of the study outcome (expected time for return to work versus actual return to work after 6 months), cross-sectional study type, larger sample size and less covariables included in the adjusted model may possibly explain higher estimates and statistical significance of these results. Nevertheless, both studies are consistent in finding negative associations of ERI with return to work.

Overcommitment, which represents an independent factor that is not included in the ERI score, was also considered in a few studies. The association of high overcommitment with higher risk of cardiovascular diseases was reported [41]. Furthermore, overcommitment seemed to be associated with lower quality of life [8], higher sick-leave level [42] and low return to work self-efficacy in persons with mental disorders [43]. The average overcommitment score was 7.2 in a study on patients with recurrent heart diseases [16], which is only one half of the score found the present study. Since the mentioned studies did not only focus on AMI patients, comparability is restricted. Association of overcommitment with return to work in patients with AMI was not investigated so far. The results of this study underline the importance of overcommitment as an associated factor of return to work.

The study findings indicate that work-related stress, as assessed by ERI, and overcommitment may be associated with return to work. This finding is relevant for the patients since these risk factors of work disability are shown to be modifiable [44]. Reduction of ERI may not only positively affect the patients’ quality of life [45, 46], but also hard clinical outcomes such as the risk of recurrent AMI [16, 47]. Thus, a reduction of stressors at the workplace or an improvement of coping with work-related stress may even improve survival post-AMI.

The covariables DM and current smoking showed strong associations (OR of 2.48–2.59 for DM and 3.25–3.46 for current smoking) with no return to work. Other studies investigating DM and return to work showed varying results. Mustafah et al. [37] detected that DM was positively related with return to work after cardiac events. In contrary, another study revealed positive associations of DM with retirement from work after AMI in a cohort of more than 39,000 people, even though OR was only 1.30 in the fully-adjusted model [36]. Adjustment for varying covariables in the models may account for the different results, e.g. Mustafah et al. [37] adjusted for HRQOL, whereas Smedegaard et al. [36] did not. Both studies, however, did not adjust for work stress-related variables such as the ERI score.

The association between current smoking and return to work may be explained by the observation that smoking is an indicator of the socioeconomic status. Smoking differs significantly between socioeconomic groups, with high consumption in groups with low socioeconomic status and low consumption in groups with high socioeconomic status [48]. Furthermore, socioeconomic status is associated with later recovery after AMI [49], explaining associations between low socioeconomic status and no return to work at 6-months follow-up after an AMI. Even though no association of smoking with return to work was found in cohorts of patients with musculosceletal diseases [50], a study on patients with AMI showed significant associations between current or ex-smoking and no return to work [38], corresponding to results that were obtained in the present study.

The significant association between low physical and mental HRQOL, and no return to work, which was found in the present study is coherent with findings from other studies. Mehrdad et al. [51] detected significant associations between physical components of HRQOL and return to work in a group of patients 3 months after coronary artery bypass surgery. Physical, mental and overall HRQOL were found to be significantly higher in patients who returned to work after AMI (2.20 to 1.94 mean scores for overall HRQOL) and lower in patients who did not return to work [52]. This effect achieved statistical significance in unadjusted and adjusted models. In conclusion, physical and mental HRQOL seem to have an important influence on return to work in patient groups with cardiac diseases, especially with AMI.

### Strengths and Limitations

To our knowledge, this is the first study to investigate the association between work-related stress and return to work after an AMI. The study has a longitudinal design which enables follow-up of a certain sample at several points of time. A considerable number of variables covering physical and mental health, psychosocial and stress-related factors as well as clinical factors were assessed and selected for the logistic regression model by methodologically sound techniques. Also, data from a disease registry was used, ensuring standardized data collection procedures and good data quality.

A sensitivity analysis provided similar results for the three models of the logistic regression analysis after stratifying for certified sick people in the group of no return to work. Thus, it can be assumed that the results are not depending on the reason for no return to work. Furthermore, a selection bias may have led to an underestimation of the strength of the association between independent variables and outcome, since a number of participants had missing data for the required variables and were excluded from regression analyses. Compared with the participants with available information on all variables, significantly higher rates of overcommitment, DM, perceived stress, financial burden, stress at work and lower rates of HRQOL were found.

However, missing data of covariables led to a considerable reduction of the sample size for the logistic regression analysis and a loss of statistical power. Another limitation of the study is the lack of a specific assessment of the socioeconomic status of the study sample.

The results of the present study indicated that personal factors such as work-related stress and overcommitment may influence return to work, but the associations were not significant. The association between resilience and return to work was slightly stronger and borderline significant. In contrast, HRQOL, specifically the mental dimension, was strongly associated with return to work and appears to be more important than work-related stress and also clinical factors, such as AMI classification, which do not play a major role as determinants of return to work after AMI. In order to improve the patients’ ability to cope with stressful work, rehabilitation programs should focus on psychological interventions for patients who report poor HRQOL or resilience. Some guidelines already recommend validated psychosocial screening programs [53]. Inclusion of questionnaires regarding HRQOL and resilience into screening programs for female and male patients should become standard. Legal recognition and appropriate financial support of targeted intervention programs would facilitate return to work after an AMI.
