# Correction to: Effort-Reward Imbalance at Work and Overcommitment in Patients with Acute Myocardial Infarction (AMI): Associations with Return to Work 6 Months After AMI

**DOI:** 10.1007/s10926-021-09996-1

**Published:** 2021-08-06

**Authors:** Sarah Ruile, Christine Meisinger, Katrin Burkhardt, Margit Heier, Christian Thilo, Inge Kirchberger

**Affiliations:** 1 Chair of Epidemiology, UNIKA-T Augsburg, Ludwig-Maximilians-Universität München, Neusässer Str. 47, 86156 Augsburg, Germany; 2 Institute for Medical Information Processing, Biometry and Epidemiology-IBE, Ludwig-Maximilians-Universität München, Munich, Germany; 3 Pettenkofer School of Public Health, Munich, Germany; 4 MONICA/KORA Myocardial Infarction Registry, University Hospital of Augsburg, Augsburg, Germany; 5 Independent Research Group Clinical Epidemiology, Helmholtz Zentrum München, German Research Center for Environmental Health, Neuherberg, Germany; 6 Department of Laboratory Medicine and Microbiology, University Hospital of Augsburg, Augsburg, Germany; 7 Institute of Epidemiology, Helmholtz Zentrum München, German Research Center for Environmental Health, Neuherberg, Germany; 8 KORA Study Centre, University Hospital of Augsburg, Augsburg, Germany; 9 Department of Internal Medicine I – Cardiology, University Hospital of Augsburg, Augsburg, Germany; 10 Centro de Investigación Biomédica en Red, Enfermedades Cardiovasculares (CIBERcv), Madrid, Spain

## Correction to: Journal of Occupational Rehabilitation 10.1007/s10926-020-09942-7

The article Review of Effort‑Reward Imbalance at Work and Overcommitment in Patients with Acute Myocardial Infarction (AMI): Associations with Return to Work 6 Months After AMI, written by Inge Kirchberger, was originally published electronically on the publisher’s internet portal on 12 November 2020 without open access. With the author(s)’ decision to opt for Open Choice the copyright of the article changed on 9 July 2021 to © The Author(s) 2021 and the article is forthwith distributed under a Creative Commons Attribution.

